# Identification of correlative factors and development of a preliminary classification model for delirium superimposed on dementia: a cross-sectional study

**DOI:** 10.3389/fpsyt.2026.1889440

**Published:** 2026-09-16

**Authors:** Simin Liu, Yu Xu

**Affiliations:** West China Hospital, Sichuan University, Chengdu, China

**Keywords:** classification model, correlative factors, cross-sectional study, delirium, dementia

## Abstract

**Objective:**

This study aimed to identify the correlative factors of delirium in patients with dementia and to preliminarily develop a classification model for probable delirium superimposed on dementia (DSD).

**Methods:**

A total of 143 dementia inpatients from a tertiary hospital were enrolled. Participants were assessed using the Mini-Mental State Examination (MMSE) to evaluate baseline cognitive status, and the Confusion Assessment Method-Chinese Revision (CAM-CR) was applied to categorize participants into the probable delirium group (CAM-CR > 19) and the non-probable delirium group (CAM-CR ≤ 19). Assessments of clinical characteristics included the Generalized Anxiety Disorder Seven-Item Scale (GAD-7), 30-Item Geriatric Depression Scale (GDS-30), Nurses’ Global Assessment of Suicide Risk (NGASR), Social Support Rating Scale (SSRS), Barthel Index of Activities of Daily Living (ADL), and Brøset Violence Checklist (BVC). Independent-samples *t*-tests, chi-square tests, and binary logistic regression were used to identify correlative factors and to develop the classification model. Model performance was evaluated using the area under the receiver operating characteristic curve (AUC).

**Results:**

The prevalence of CAM-CR-assessed probable delirium was 40.6%. Binary logistic regression identified three independent correlative factors of probable delirium in patients with dementia: the number of comorbid physical diseases [odds ratio (OR) = 1.458, 95% confidence interval (CI): 1.121–1.897], level of social support utilization (OR = 0.833, 95% CI: 0.703–0.987), and risk level of violence (medium risk: OR = 4.677, 95% CI: 1.373–15.929; high risk: OR = 13.073, 95% CI: 3.560–48.002). The final classification model, visualized via a nomogram, demonstrated an apparent AUC of 0.767 (95% CI: 0.689–0.844). Bootstrap validation (1,000 iterations) yielded an optimism-corrected AUC of 0.744 (bias = 0.023), and fivefold cross-validation produced a mean AUC of 0.735 (SD = 0.113). The calibration curve showed good agreement between predicted and observed probabilities (mean absolute error = 0.024). The Hosmer–Lemeshow test indicated good calibration (*χ*^2^ = 3.477, *p* = 0.901).

**Conclusion:**

The number of comorbid physical diseases, utilization of social support, and violence risk were significant correlative factors of probable delirium in dementia inpatients. The nomogram represented a proof-of-concept classification tool for DSD screening. However, given the limitations of the study design, sample size, and the absence of external validation, this model should be regarded as a preliminary tool that requires external validation before clinical application.

## Introduction

Dementia is a progressive clinical syndrome characterized by a severe decline in cognitive function and activities of daily living (1). It results from irreversible brain damage; meanwhile, delirium is typically an acute condition with a variable duration and fluctuating course (2). Delirium occurring in a patient with preexisting dementia is referred to as delirium superimposed on dementia (DSD). Recent studies indicate that delirium superimposed on preexisting dementia is highly prevalent in the geriatric inpatient population, with a pooled prevalence of 9.7%–48.9% (3). DSD leads to critical outcomes, such as increased mortality and higher rates of institutionalization (4). By accelerating the underlying dementia trajectory, DSD results in impaired functional outcomes, higher institutionalization and readmission rates, and increased mortality; thus, early detection and prevention are crucial (5).

DSD presents significant diagnostic challenges because key delirium indicators such as inattention, cognitive dysfunction, and altered consciousness might substantially overlap with core dementia symptoms (6). Only approximately 35% of delirium cases are clinically recognized because clinicians might attribute delirium symptoms to underlying dementia or acute mental illness, especially in hypoactive delirium manifestations (7). Even for healthcare providers who initially notice mental status changes in patients with dementia, the lack of specific evaluation methods hinders the early detection of DSD, or such changes might be generally labeled as sundowning syndrome (8, 9). Therefore, awareness of delirium and DSD among frontline medical professionals remains limited (10). DSD is associated with significantly more severe outcomes than delirium or dementia alone, including higher rates of mortality, institutionalization, prolonged hospitalization, and accelerated cognitive decline (11). Notably, untreated DSD can lead to profound functional deterioration by impairing consciousness, cognition, psychomotor activity, and sleep-wake cycles, thereby substantially reducing independence in older adults (12).

The concealment and complexity of DSD present diagnostic challenges, particularly in severe cases because of the overlap between delirium and dementia symptoms (13). Previous DSD diagnostic methods have focused on laboratory and biological indicators, including biomarkers in cerebrospinal fluid and blood, neuroimaging, and electroencephalography (14, 15). Additionally, prior studies reported that caregiver burden and social support might have bidirectional impacts on the occurrence of DSD (16, 17). Given the complex nature of DSD, its risk assessment should be multidimensional. Current DSD evaluation models were developed according to general demographic information, living conditions, and biochemical laboratory indicators; however, psychological factors, such as depressive symptoms and anxiety, have rarely been included, despite their reported associations with delirium among patients with dementia (18, 19). Therefore, by including psychological and social factors, this study aimed to develop a brief and applicable DSD screening tool for patients with dementia.

## Method

### Study population

In this cross-sectional study, demographic data, psychosocial scale assessments, and Confusion Assessment Method-Chinese Revision (CAM-CR) evaluations were all collected concurrently within 3 days after admission; therefore, temporal precedence between predictors and the outcome could not be established. Inclusion criteria were as follows: (1) with a diagnosis of dementia in accordance with the International Classification of Diseases 10 (ICD-10); and (2) the patient or the patient’s legal guardian agreeing to sign informed consent. Exclusion criteria were as follows: (1) participants suffering from end-stage diseases (with a life expectancy of <6 months); (2) participants with hearing or visual impairment that might hinder communication; and (3) participants who were unwilling to complete the investigation for various reasons. Participants were allocated into two groups based on CAM-CR scores: non-probable delirium (CAM-CR score ≤ 19) and probable delirium (CAM-CR score > 19) (20).

### Sample size estimation

The sample size was initially calculated for a cross-sectional survey using the conventional rule of 5–10 times the number of variables (21 variables) for descriptive analyses. Accounting for a potential 20% attrition/invalid response rate, 132–263 participants were required. The final multivariable model contained four regression parameters: number of comorbid physical diseases (*n* = 1), utilization of social support (*n* = 1), and dummy-coded indicators for Brøset Violence Checklist (BVC) violence risk (*n* = 2). The events-per-parameter ratio was 58/4 = 14.5, exceeding the recommended minimum of 10 events per parameter (21). The total of 143 participants met the predetermined sample size criteria for the descriptive objectives, and the EPP for the final model was adequate. Nevertheless, the risk of model overfitting should be noted, and the findings should be considered preliminary pending validation in larger cohorts.

### Data collection

Data collection was completed within 3 days after admission. Given the potential for cognitive deficits to compromise the accuracy of self-reports in patients with dementia, physicians initially evaluated clinical stability before assessment. Individuals with clinical stability and adherence to medical treatment were enrolled for questionnaire completion. Notably, the 30-Item Geriatric Depression Scale (GDS-30) and Generalized Anxiety Disorder Seven-Item Scale (GAD-7) in this study are self-report scales, and patients with dementia may have diminished insight due to cognitive deficits, which could compromise the accuracy of their self-reported psychological symptoms. To mitigate this limitation, appropriate measures were applied to improve response quality: research assistants had completed standardized training in assisting eligible participants with completing self-report scales. Interviewers read and explained the items to participants who had difficulty understanding them. When participants could not provide a clear response to specific items, their primary caregiver was consulted for verification, and the caregiver-confirmed response was recorded. After the interview, research assistants collected the questionnaire and checked them for completeness. In addition, there were no missing data in the final analysis because incomplete items were checked on-site and completed immediately.

### Questionnaire

#### The General Information Questionnaire

A structured questionnaire was used to collect general demographic information from participants, including gender, age, marital status, education level, total duration of hospitalization, and other clinical characteristics.

#### The Mini-Mental State Examination

The Mini-Mental State Examination (MMSE) was used to characterize the baseline cognitive status of participants. Developed by Folstein et al. in 1975, the MMSE is a widely used 30-point screening tool that evaluates orientation, memory, attention, language, and visuospatial abilities. Scores range from 0 to 30, with lower scores indicating greater cognitive impairment. The Cronbach’s α of the MMSE in this study was 0.719 (22).

#### Confusion Assessment Method-Chinese Revision

CAM-CR is a widely used Chinese-adapted auxiliary diagnostic tool reflecting clinical delirium status. CAM-CR assesses multiple domains, including impaired attention, psychomotor retardation, acute onset, and memory decline. Total scores range from 0 to 44, and a CAM-CR > 19 indicates probable delirium (23). The Cronbach’s α of the CAM-CR in this study was 0.735.

#### Generalized Anxiety Disorder Seven-Item Scale

GAD-7 is a brief self-administered screening tool for assessing the severity of generalized anxiety disorder. The instrument consists of seven items that evaluate the frequency of anxiety symptoms over the past 2 weeks, including feeling nervous or anxious, inability to control worrying, excessive worrying, difficulty relaxing, restlessness, irritability, and feelings of being afraid. Each item was rated on a 4-point Likert scale with a total score ranging from 0 to 21. Scores of 5, 10, and 15 represented cutoff points for mild, moderate, and severe levels of anxiety, respectively (24). The Cronbach’s α of the GAD-7 in this study was 0.881.

#### Thirty-Item Geriatric Depression Scale

The Chinese version of GDS-30 is used to identify unique symptoms of geriatric depression (25). The total score of the GDS-30 ranges from 0 to 30, with higher scores indicating a more severe degree of depression. The scale indicates good internal consistency, and the GDS has been extensively validated across different populations in China (26). The Cronbach’s α of the GDS-30 in this study was 0.929.

#### Nurses Global Assessment of Suicide Risk

The Nurses’ Global Assessment of Suicide Risk (NGASR), developed by Cutcliffe and Barker in 2004, is applied to measure the risk of suicide. This scale comprises 15 items, each assigned a specific value based on its relevance to suicide risk. NGASR scores range from 0 (low level of suicide risk) to 25 (high level of suicide risk). The Cronbach’s α of the NGASR in this study was 0.751 (27).

#### Social Support Rating Scale

The 10-item Social Support Rating Scale (SSRS), developed by Shuiyuan Xiao in 1986, includes three dimensions: objective support, subjective support, and utilization of social support. SSRS scores range from 12 to 66: less than 22 indicates a low level of social support, 23 to 44 indicates a medium level of social support, and ≥ 45 indicates a high level of social support (28). The social support utilization sub-dimension (items 8–10 of the SSRS) assesses three aspects: (a) the frequency of active participation in social, community, or peer-group activities (scored from 1 = never to 4 = very often); (b) the tendency to seek help when encountering difficulties (scored from 1 = never seeks help to 4 = always actively seeks help); and (c) willingness to attend peer support gatherings if applicable (scored from 1 = never attends to 4 = actively attends). Lower total scores of this subdimension indicate a lower level of social support utilization. The Cronbach’s α of the SSRS in this study was 0.777.

#### The Barthel Index of Activities of Daily Living

The Barthel Index (BI) of Activities of Daily Living is an ordinal scale designed to measure functional independence in activities of daily living. Developed by Barthel et al. in 1965, this instrument assesses 10 domains: feeding, bathing, grooming, dressing, bowel control, bladder control, toilet use, transfers (bed to chair), mobility on level surfaces, and stair climbing. Each item is scored based on the degree of assistance required, with the original version using increments of 5 points. Total scores on the BI range from 0 to 100, with higher scores indicating greater independence and lower scores reflecting increased functional disability (29). The Cronbach’s α of BI in this study was 0.904.

#### The Brøset Violence Checklist

The BVC is a brief, six-item risk assessment tool for predicting imminent violent behavior. The level of risk of violence is assessed based on the presence of the following behavioral indicators: confusion, irritability, boisterousness, verbal threats, physical threats, and attacks on objects. Each present behavior is scored as 1 point; a total score of 0 indicates a low level of risk of violence, a score of 1–2 indicates a moderate level of risk requiring preventative measures, and a score of 3 or above indicates a high level of risk necessitating immediate intervention (30). The Cronbach’s α of BVC in this study was 0.769.

### Statistical analysis

SPSS 25.0 and R v4.5.2 were used for data analysis. Normally distributed quantitative data were presented as mean ± standard deviation. Count data were presented using frequency and proportion. Independent-samples *t*-tests and chi-square tests were conducted to identify statistically significant factors. Variables with *p* < 0.05 in univariate analysis were entered into the multivariable logistic regression model, and only variables that remained statistically significant were retained. The area under the receiver operating characteristic curve (AUC) was used to evaluate the discriminative ability of the model. Bootstrap validation with 1,000 iterations, fivefold cross-validation, and calibration curve analysis were performed. The Hosmer–Lemeshow test was performed to assess the calibration of the model.

## Result

### Demographic and clinical variables

A total of 143 participants with dementia were recruited between October 2024 and December 2025. **Table 1** displays the clinical characteristics of participants and comparisons of relevant demographic features between the probable delirium group and the non-probable delirium risk group. The mean age of participants was 75.62 ± 9.13 years, with a range of 59–97 years. Among the 143 participants, 93 (65.0%) were women. Apart from the number of comorbid physical diseases and MMSE scores, no statistically significant differences were observed between the probable delirium group and the non-probable delirium group in gender, age, education level, marital status, first-time admission status, admission route, physical restraint use, disease duration, psychotropic medication use, and total duration of hospitalization.

**Table 1:** Demographic and clinical characteristics of dementia inpatients with and without probable delirium (*n* = 143).

Variables	Categories	Total (*n* = 143)	Non-probable delirium (*n* = 85)	Probable delirium (*n* = 58)	*x* ^2^ */F/t*	*p*-value
Gender	Man	50 (35.0)	28 (32.9)	22 (37.9)	0.377	0.539
	Woman	93 (65.0)	57 (67.1)	36 (62.1)		
Age (years; M ± SD)		75.62 ± 9.13	74.67 ± 9.153	77.02 ± 9.00	1.516	0.132
Education	Primary and below	54 (38.3)	30 (36.1)	24 (41.4)	5.049	0.080
	Middle school	48 (34.0)	26 (31.3)	22 (37.9)		
	Senior high school and above	39 (27.7)	27 (32.6)	12 (20.7)		
Marital status (have a spouse)	Yes	106 (74.1)	58 (68.2)	48 (82.8)	3.791	0.052
	No	37 (25.9)	27 (31.8)	10 (17.2)		
First admission	Yes	98 (68.5)	59 (69.4)	39 (67.2)	0.075	0.784
	No	45 (31.5)	26 (30.6)	19 (32.8)		
Emergency admission	Yes	34 (23.8)	19 (20.2)	15 (25.9)	0.234	0.628
	No	109 (76.2)	66 (64.8)	43 (74.1)		
Number of comorbid physical diseases (M ± SD)		2.48 ± 1.53	2.25 ± 1.39	2.81 ± 1.67	**2.193**	**0.030**
Physical restraints	Yes	11 (7.7)	5 (5.9)	6 (10.3)	–	0.353
	No	132 (92.3)	80 (94.1)	52 (89.7)		
Course of dementia (M ± SD)		5.23 ± 4.32	5.30 ± 3.86	5.18 ± 4.63	0.156	0.876
Psychotropic medication use	Yes	119 (83.2)	70 (82.4)	49 (84.5)	0.112	0.738
	No	24 (16.8)	15 (17.6)	9 (15.5)		
Hospitalization (days; M ± SD)		26.11 ± 16.40	24.52 ± 15.37	28.45 ± 17.67	1.412	0.160
MMSE score (M ± SD)		11.20 ± 7.01	13.78 ± 6.93	7.43 ± 5.22	5.039	**< 0.001**

Bold values indicate statistical significance (P < 0.05).

The proportion of participants with dementia with probable delirium was 40.6% (58/143). The results indicated statistically significant differences in various measures: among psychological factors, the GDS-30 scores were higher in participants with dementia with probable delirium than in those without probable delirium (**Table 2**). As shown in **Table 3**, the scores for the “Utilization of social support” subdimension of the SSRS and BI scores in the group without probable delirium were higher than those in the group with probable delirium. In contrast, the BVC score in the group without probable delirium was lower than that in the group with probable delirium.

**Table 2:** Comparison of psychological health status between dementia patients with and without probable delirium (*n* = 143).

Variables		Total (*n* = 143)	Non-probable delirium (*n* = 85)	Probable delirium (*n* = 58)	*χ^2^/t*	*p*
GAD-7, M±SD		5.02±4.29	4.53±3.79	5.74±4.87	1.595	0.114
Level of anxiety	Normal (GAD-7≤4)	75(52.4)	50(58.8)	25(43.1)	3.905	0.142
	Mild (GAD-7, 5~9)	44(30.8)	24(28.3)	20(34.5)		
	Moderate and above (GAD-7≥10)	24(16.8)	11(12.9)	13(22.4)		
GDS-30, M±SD		13.43±7.53	12.16±7.34	15.28±7.50	**2.468**	**0.015**
Risk of depression	GDS-30≤410	59(41.3)	40(47.1)	19(32.8)	2.909	0.088
	GDS-30≥11	84(58.7)	45(52.9)	39(67.2)		
NGASR, M±SD		2.99±2.59	2.95±2.73	3.03±2.38	0.184	0.854
Risk of suicide	Low (NGASR≤405)	122(85.3)	73(85.9)	49(84.5)	0.448	0.869
	Medium (NGASR, 6~8)	15(10.5)	8(9.4)	7(12.1)		
	High (NGASR≥9)	6(4.2)	4(4.7)	2(3.4)		

Bold values indicate statistical significance (P < 0.05).

**Table 3:** Comparison of psychosocial and functional status between dementia patients with and without probable delirium (*n* = 143).

Variables			Total (*n* = 143)	Non-probable delirium (*n* = 85)	Probable delirium (*n* = 58)	*χ2/t*	*p*
SSRS (Total score), M±SD			30.18±7.61	31.69±7.94	27.97±6.56	**-2.953**	**0.004**
SSRS (Objective social support), M±SD			8.36±2.61	8.67±2.99	7.91±1.86	-1.713	0.089
SSRS (Subjective social support), M±SD			16.76±4.17	17.28±4.51	16.00±3.52	-1.819	0.071
SSRS (Utilization of social support), M±SD			5.31±2.46	5.74±2.59	4.67±2.11	**-2.710**	**0.008**
BI, M±SD			62.69±29.09	71.24±27.01	50.17±27.64	**-4.535**	**<0.001**
Level of activity function	Non-impaired (BI=100)		17(11.9)	17(20.0)	0(0.0)	**17.959**	**<0.001**
	Mildly impaired (BI, 99~61)		63(44.1)	40(47.1)	23(39.7)		
	Moderately impaired (BI, 60~41)		26(18.2)	12(14.1)	14(24.1)		
	Severely impaired (BI≤40)		37(25.9)	16(18.8)	21(36.2)		
BVC, M±SD			1.87±1.56	1.44±1.44	2.50±1.54	**4.248**	**<0.001**
Risk of violence		Low risk (BVC=0)	30(21.0)	26(30.6)	4(6.9)	**19.180**	**<0.001**
		Medium risk (BVC, 1~2)	69(48.2)	43(50.6)	26(44.8)		
		High risk (BVC≥3)	44(30.8)	16(18.8)	28(48.3)		

Bold values indicate statistical significance (P < 0.05).

### Construction of a classification model

Binary logistic regression analysis was performed on the demographic and clinical characteristics of participants with dementia to construct a classification model for probable delirium. Based on the results of the logistic regression analysis, a classification model was built. The following formula was used: logit(P) = − 2.013 + 0.377 * number of comorbid physical diseases + (− 0.183) * score of social support utilization + 2.571 * risk of violence (high risk) + 1.543 * risk of violence (medium risk) (**Table 4**). All variance inflation factors ranged between 1.019 and 1.787, which were below the threshold of 5. An additional sensitivity analysis, excluding BVC from the model, was performed. The results of this analysis showed that two variables (comorbid physical diseases and social support utilization) remained statistically significant; the model yielded *χ*^2^ = 19.74, *df* = 2, *p* < 0.001, with an AUC of 0.659 (95% confidence interval [CI]: 0.569–0.749).

**Table 4:** Binary logistic regression model for inpatients with probable delirium (*n* = 143).

Variables	B	SE	Wald	*df*	*p*-value	OR	95% CI of Exp(B)
Comorbid physical diseases	0.377	0.134	7.908	1	0.005	1.458	1.121–1.897
SSRS (utilization of social support)	− 0.183	0.087	4.451	1	0.035	0.833	0.703–0.987
Risk of violence (high risk)	2.571	0.664	14.997	1	< 0.001	13.073	3.560–48.002
Risk of violence (medium risk)	1.543	0.625	14.459	1	0.014	4.677	1.373–15.929
Constant	− 2.013	0.807	6.227	1	0.013	0.134	–

According to the results of a binary logistic regression analysis, the nomogram function in the “rms” package in R was used to construct a nomogram (**Figure 1**). **Figure 2** shows that the AUC of this model was 0.767 (95% CI: 0.689–0.844), which was notably higher than that of the model excluding BVC. Bootstrap validation (1,000 iterations) yielded an optimism-corrected AUC of 0.744 (bias = 0.023). The calibration curve showed good agreement (mean absolute error = 0.024) (**Figure 3**). The fivefold cross-validation produced a mean AUC of 0.735 (SD = 0.113) (**Figure 4**). The Hosmer–Lemeshow test result (*χ*^2^ = 3.477, *p* = 0.901) indicated that the model had good calibration. Exploratory analysis using the optimal cutoff value was performed; the Youden index identified a probability cutoff of 0.506, corresponding to a nomogram total score of 160 points. At the optimal cutoff, the sensitivity was 0.603 (95% CI: 0.475–0.719), specificity was 0.800 (95% CI: 0.703–0.871), positive predictive value (PPV) was 0.673 (95% CI: 0.538–0.785), and negative predictive value (NPV) was 0.747 (95% CI: 0.649–0.825).

**Figure 1:**
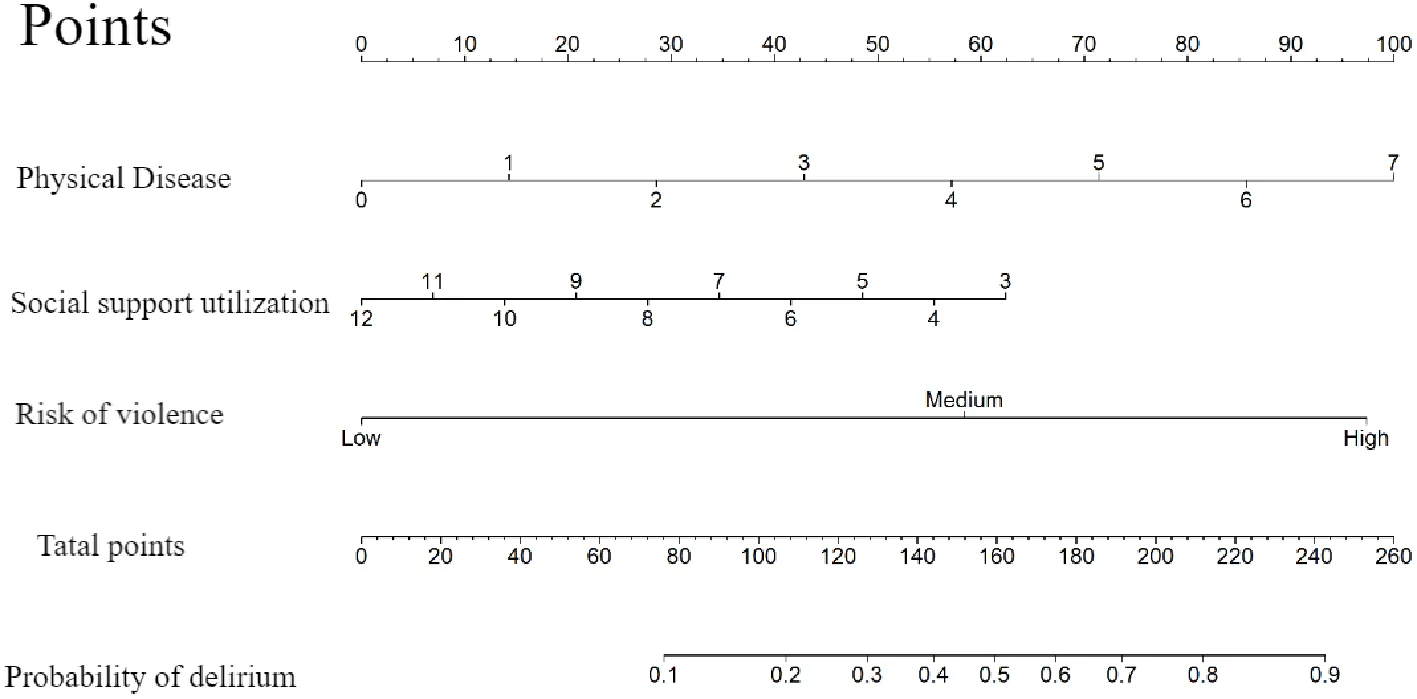
Probable delirium classification nomogram.

**Figure 2:**
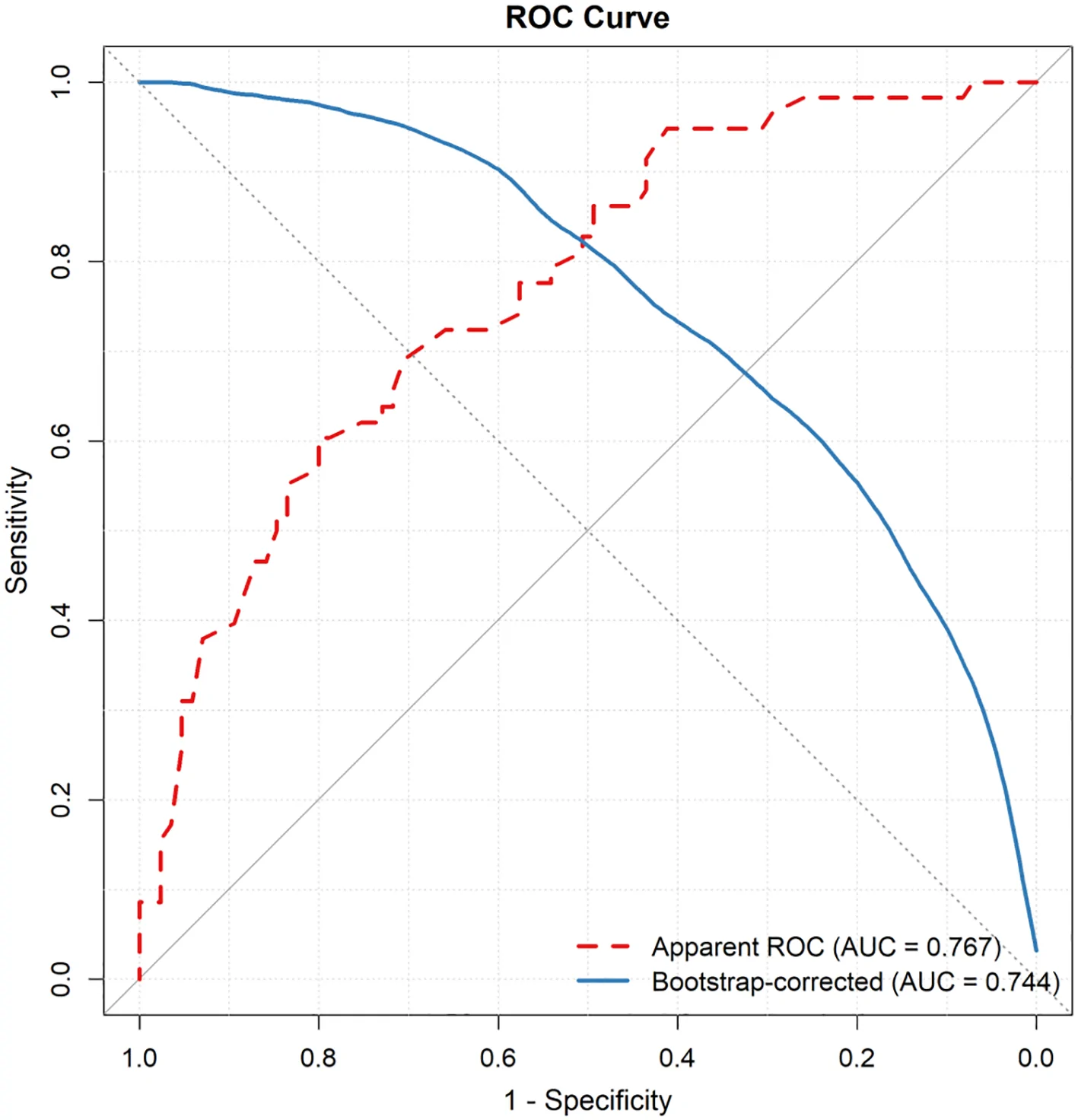
AUC of the probable delirium classification model.

**Figure 3:**
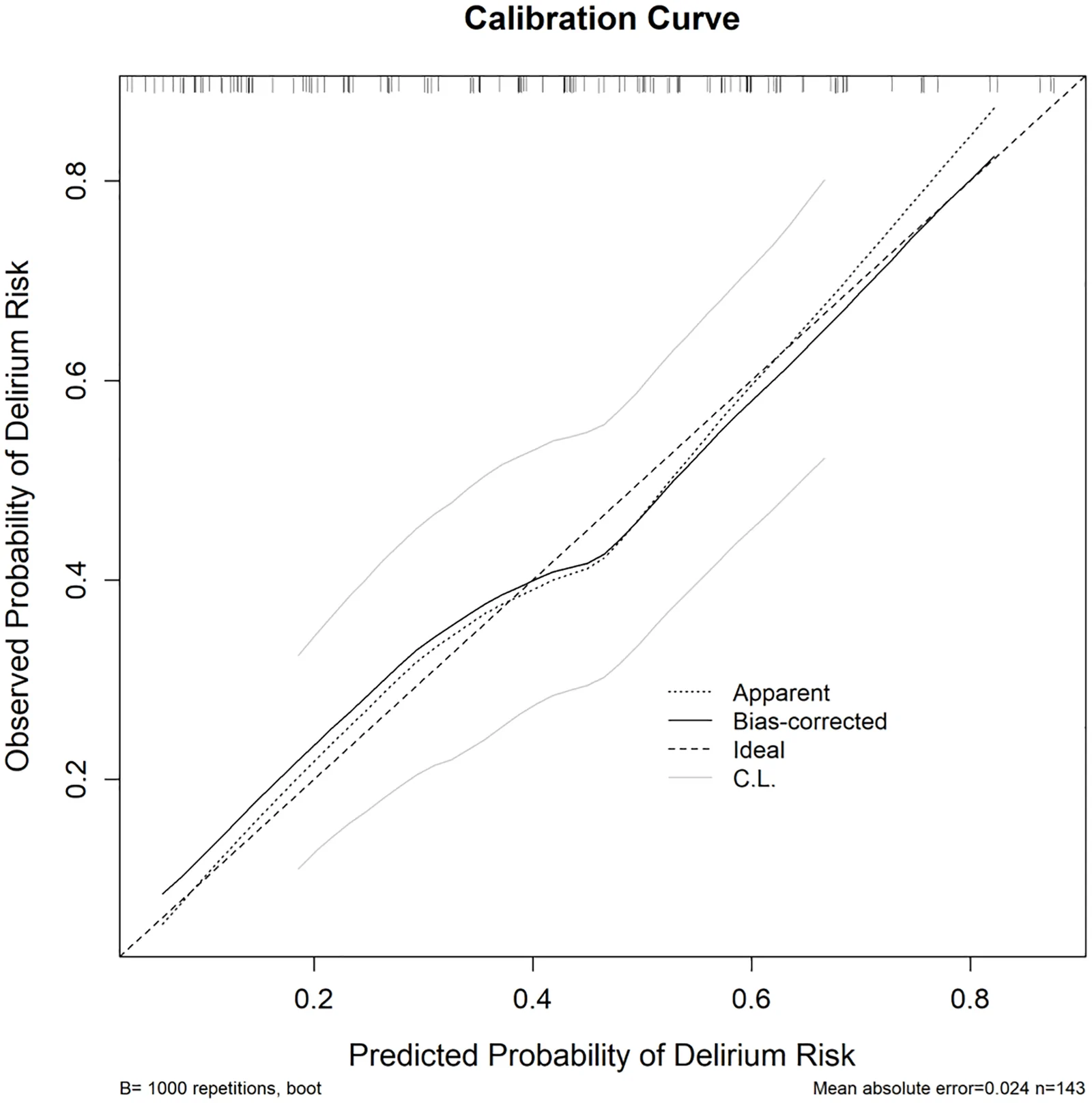
Calibration curve of the classification model (1,000 bootstrap iterations).

**Figure 4:**
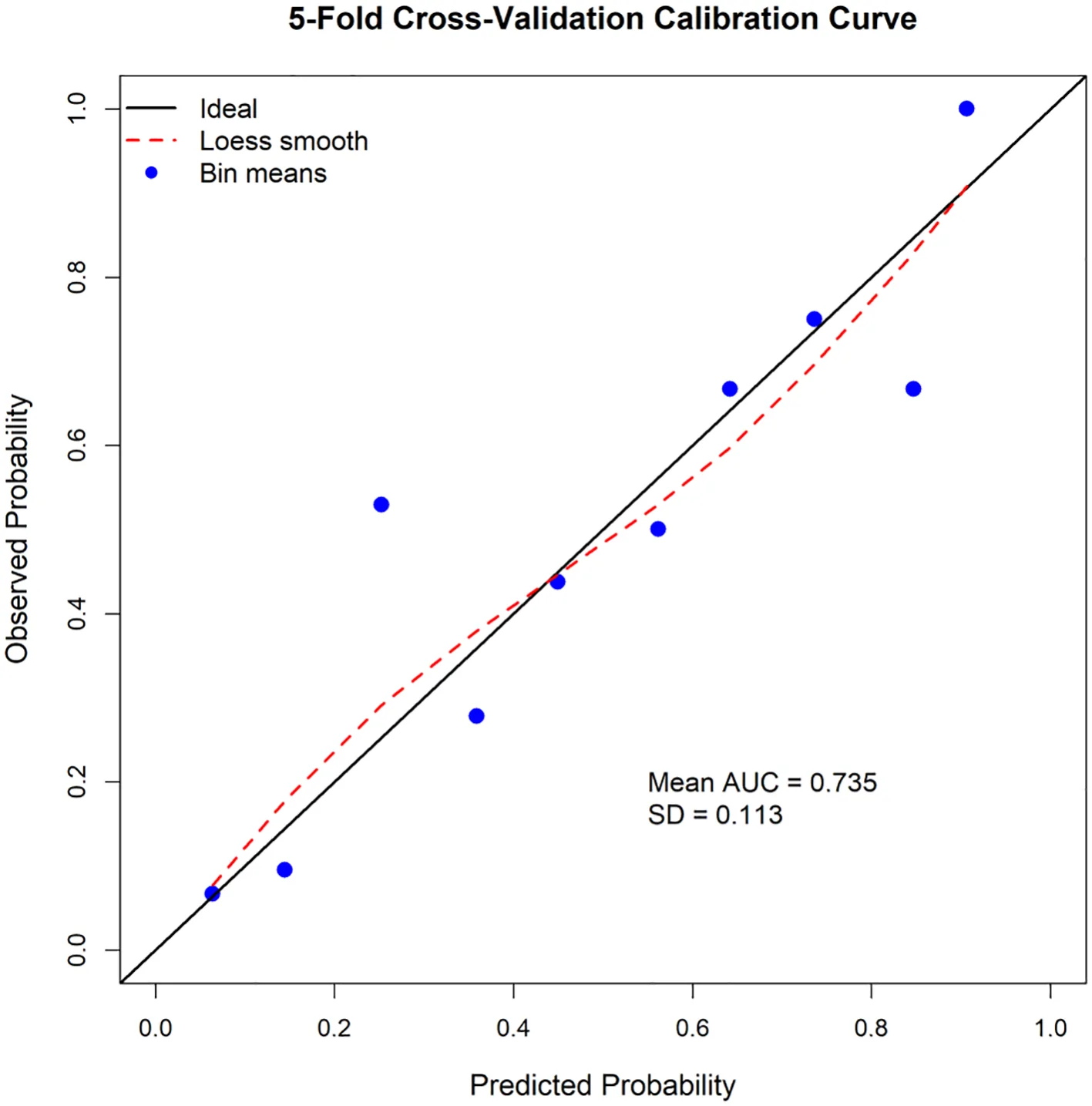
Calibration curve of the fivefold cross-validation.

## Discussion

This study investigated the prevalence of probable delirium in patients with dementia and compared the psychological characteristics and functional status between patients with dementia with and without probable delirium. In the proposed study, 40.6% of patients with dementia had probable delirium, which was similar to the findings reported by Xiao et al. (2023) (14). Our findings revealed that the number of comorbid somatic diseases, the utilization of social support, and the risk of violence were correlative factors of probable delirium in patients with dementia. The AUC of the classification model for DSD in this study was 0.767 (95% CI: 0.689–0.844), indicating moderate discrimination. The required data were readily accessible through observation, suggesting the model’s potential utility as a screening tool. However, given the wide confidence interval and the absence of external validation, the model should be considered a preliminary tool at this stage.

This study demonstrated that the number of comorbid physical diseases was a correlative factor for probable delirium in patients with dementia. A previous retrospective study examining DSD reported that diabetes and poststroke sequelae were independent risk factors for DSD (14). Recent studies also highlighted comorbidities as key factors; this might be due to multiple comorbidities exacerbating the cerebral burden (31). Generally, preexisting neurodegenerative disease reduces brain resilience, increasing the risk of delirium. Metabolic diseases further disrupt cerebral energy homeostasis, consequently impairing the compensatory capacity of the dementia-affected brain (32). Additionally, peripheral and systemic inflammation disrupts the blood–brain barrier, thereby affecting central nervous system function and disease progression and increasing the risk of delirium (33). However, the relationship between comorbidities and delirium remains complex and unclear. Future research is needed to investigate specific diseases, combination effects, and individual susceptibility factors to develop individualized DSD prevention strategies.

Currently, few studies have examined the relationship between psychological states and delirium in patients with dementia, although existing studies have confirmed that patients with higher levels of anxiety and helplessness have a greater risk of developing delirium (19, 34). In this study, although univariate analysis revealed significant differences in GDS-30 scores between patients with dementia with and without probable delirium, this variable did not emerge as a key factor in the final multivariate classification model. This difference might be due to limitations in the validity of the GDS-30 in measuring the severity of depression in patients with dementia (35, 36). Patients with dementia suffer from cognitive deficits, and diminished insight might compromise the accuracy of psychological self-report measures, leading to substantial discrepancies between patient self-reports and objective clinical assessments (37). Despite the difficulties in using psychological status to evaluate delirium risk in patients with dementia, psychological comorbidities in patients with delirium should be a concern for clinicians, particularly regarding the adoption of easy-to-use and effective identification tools (38). In addition, previous findings showed that violent behavior was associated with delirium in patients with dementia (39). Notably, BVC items (for instance, confusion, irritability, and verbal/physical threats) might also represent acute symptoms of delirium. This overlap should be interpreted as indicating that the BVC score might reflect concurrent manifestations of delirium. Despite the causal direction between the BVC score and delirium remaining unclear, when patients with dementia exhibit aggressive behavior or are in high-risk situations for violence, medical professionals should recognize that such aggression might indicate the possible occurrence of delirium, rather than representing general abnormal behavior (40).

Limited evidence has explored the association between social support and DSD. Blotenberg et al.(2024) found that a low level of social support was strongly associated with adverse outcomes in patients with dementia, such as depression and mortality risk (41). However, a study by Lehmann et al.(2025) in Colombia found no significant associations between social determinants of health (including living arrangements and social support indicators) and delirium in older hospitalized patients (42). In addition, empirical studies propose that declining activities of daily living function are strongly associated with increased delirium risk in patients with dementia (43). Our results showed that a low level of social support utilization was associated with probable delirium, and several possible interacting pathways should be noted: a low level of social support utilization may lead to poor medication adherence and inadequate management of chronic conditions (44); this may then contribute to malnutrition and dehydration, both established delirium precipitants (45); and chronic psychosocial stress may activate the HPA axis, resulting in elevated cortisol levels that impair hippocampal function and reduce cognitive reserve (46). However, given the cross-sectional design, our study could not determine whether declining social support utilization predisposes patients to delirium or results from early cognitive or behavioral changes. Thus, further prospective studies with repeated assessments would be necessary to clarify causal pathways. Both Barthel Index of Activities of Daily Living (ADL) impairment and inadequate social support utilization represent indicators of vulnerability and dependency in patients with dementia. However, social support utilization might play an important role in identifying probable delirium in patients with dementia. First, the utilization of social support represents a dynamic, modifiable external resource that may buffer against delirium-precipitating stressors. Second, social support utilization captures environmental and psychosocial factors that are different from cognitive or physical decline (47). Currently, most DSD classification models focus on clinical and cognitive variables rather than social determinants; therefore, future research should integrate social determinants alongside traditional clinical factors to develop effective evaluation tools.

In conclusion, this study identified that the number of comorbid physical diseases, utilization of social support, and violence risk were correlative factors of probable delirium in patients with dementia, and a nomogram was accordingly developed to facilitate visual risk classification. The proposed model should be regarded as a proof-of-concept tool that requires external validation before clinical application. Clinical healthcare professionals may consider monitoring these indicators; however, the nomogram should be validated in further multicenter prospective studies. Limitations of this study should be noted: (a) CAM-CR was an assessment tool focused on probable delirium rather than a diagnosis of delirium, and the use of self-report scales (GDS-30 and GAD-7) might influence response accuracy because of potential measurement bias introduced by combining patient self-report with caregiver verification in cognitively impaired patients; (b) although the events-per-parameter ratio of 14.5 exceeded the minimum recommendation, the sample size might influence model stability and generalizability; (c) due to the cross-sectional study design, in which variables were measured concurrently, the causal relationship between the outcome and its correlative factors remained unclear. Future studies are needed to address the above concerns by applying prospective study designs, large-sample validation, and targeted assessments.

## Data Availability

The datasets presented in this article are not readily available because of privacy. Requests to access the datasets should be directed to 121043156@qq.com.
